# A case report and a literature review of drug-induced liver injury caused by Huzhang according to the updated RUCAM

**DOI:** 10.1097/MD.0000000000047385

**Published:** 2026-01-30

**Authors:** Xinyan Liang, Xiu Yang, Tianjiong Luo, Wenliang Dun

**Affiliations:** a Drug Clinical Trial Institution, Nanjing Hospital of Chinese Medicine Affiliated to Nanjing University of Chinese Medicine, Nanjing, Jiangsu Province, China; b Department of Clinical Pharmacy, Nanjing Hospital of Chinese Medicine Affiliated to Nanjing University of Chinese Medicine, Nanjing, Jiangsu Province, China; c School of Basic Medicine and Clinical Pharmacy, China Pharmaceutical University, Nanjing, Jiangsu Province, China; d Department of Gastroenterology, Nanjing Hospital of Chinese Medicine Affiliated to Nanjing University of Chinese Medicine, Nanjing, Jiangsu Province, China; e Department of Gerontology, Nanjing Hospital of Chinese Medicine Affiliated to Nanjing University of Chinese Medicine, Nanjing, Jiangsu Province, China.

**Keywords:** case report, DILI, Huzhang, Polygoni Cuspidati Rhizoma et Radix

## Abstract

**Rationale:**

Herbal medicine-induced hepatotoxicity is a significant contributing factor to drug-induced liver injury (DILI), but the mechanisms underlying herbal medicine-induced hepatotoxicity remain unclear. This study reports a well-documented probable case of DILI caused by Huzhang (Polygoni Cuspidati Rhizoma et Radix) to elucidate its clinical presentation and potential mechanisms.

**Patient concerns:**

A 72-year-old patient developed hepatocyte-type damage after continuous administration of a Huzhang-containing formula for 14 days.

**Diagnoses:**

After ruling out other causes via comprehensive exams, we reviewed the literature, excluded other medications, and applied the updated Roussel Uclaf Causality Assessment Method, which gave a score of 6 for Huzhang-induced DILI.

**Interventions:**

The patient was treated with N-acetylcysteine and other supportive medications.

**Outcomes:**

The patient’s liver function returned to normal after treatment.

**Lessons:**

The hepatotoxicity mechanism of emodin in Huzhang was explored, and it was suggested that traditional Chinese medicines containing emodin, such as Huzhang, should be cautious about hepatotoxicity risks, particularly in elderly patients, long-term users, and those with a history of surgery.

## 1. Introduction

Drug-induced liver injury (DILI) is a major adverse reaction associated with drug use, particularly in the context of traditional Chinese medicine, dietary supplements, antituberculosis drugs, and antibiotics.^[1]^ The incidence rate of DILI in China is 23.8 per 100,000 people.^[2]^ China is a major consumer of traditional Chinese herbal medicines, with DILI caused by herbal medicines or dietary supplements accounting for 26.81% of cases, and the incidence rate may continue to rise globally.^[3]^ Due to the difficulty in diagnosing DILI, multiple factors must be considered when determining which medication caused the liver injury. Here we present a case report of liver injury caused by herbal medicines, with a focus on identifying the specific medication responsible for the liver injury, providing a framework for further consideration.

## 2. Case presentation

**Figure 1.**
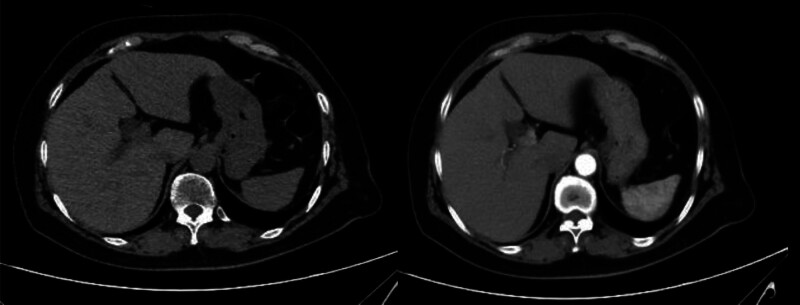
Abdominal CT scan. CT = computer tomography.

A 72-year-old female patient who had been taking traditional Chinese medicine for chronic atrophic gastritis visited the hospital for a follow-up examination on April 3, 2020. Laboratory tests revealed abnormal elevation of liver function on the same day (Table 1). Alanine aminotransferase (ALT) was 479 U/L (reference range: 8–45 U/L), exceeding the upper limit of normal by more than 5-fold, meeting 1 of the criteria for liver damage.^[4]^ She was admitted on April 4 for further treatment. The patient reported mild upper abdominal pain but no symptoms such as jaundice or hepatomegaly. She denied a history of alcohol consumption and viral hepatitis, but had a history of right shoulder surgery and cholecystectomy. After admission, as shown in Table 2, further tests were conducted. Hepatitis B surface antigen (HBsAg), hepatitis C virus antibody (HCV-IgM), cytomegalovirus antibody (CMV-IgM), and Epstein-Barr virus antibody (EBV-IgA) were all negative. Immunological tests, including antinuclear antibody (ANA), anti-smooth muscle antibody, and anti-soluble liver antigen antibody (anti-SLA), were all negative. Additionally, abdominal computer tomography (CT) showed mild dilation of the common bile duct, with the liver and pancreas appearing normal in shape and no abnormal density in the liver parenchyma (Fig. 1). Hepatitis viruses, autoimmune liver disease, and hepatobiliary-pancreatic diseases were ruled out. Upon careful inquiry into the patient’s medication history, it was found that the patient was prescribed a traditional Chinese medicine formula containing Huzhang and Muxiang on February 25, 2020, for the treatment of digestive system diseases related to liver and gallbladder damp-heat and spleen deficiency with qi stagnation. The medication was taken for 14 days and discontinued on March 9. The time interval between discontinuation of the medication and the detection of abnormal liver function was 24 days. This formula adheres to time-related principles and includes Huangqin, Yujin, Chuipencao, Muxiang, Shengma, Huzhang, Dihuang, Gancao, Baizhu, Baizhi, Huangqi, Sharen, Chaihu, and others. Based on the patient’s medication history before and after the incident, it was found that subsequent administration of Chinese herbal medicines such as Huangqin, Yujin, Chuipencao, and Dihuang did not result in liver injury. According to the updated Roussel Uclaf Causal Assessment Method (RUCAM), these herbs received a score of 2, indicating they are unlikely to cause DILI (Tables 3 and 4). However, the use of Huzhang and Muxiang was discontinued after March 9th. We have not identified any relevant literature linking Muxiang to DILI. Therefore, the updated RUCAM score is 5, indicating a possible association. For Huzhang (Anhui Huchuntang Chinese Herbal Medicine Decoction Pieces Co., Ltd., Bozhou, Anhui, China, Batch No.: 190901), the updated RUCAM score was 6, indicating a probable causal relationship (Table 3). Therefore, the diagnosis was liver injury with an R score of 9.55, consistent with the hepatocellular pattern.

**Table 1:** Liver function indicators.

Indicators date	April-3	April-8	April-13	May-23
ALT (8–45 U/L)	479	73	25	12
AST (8–45 U/L)	295	16	12	16
ALP (42–140 U/L)	156	84	76	81
GGT (7–50 U/L)	645	314	229	47
TBIL (5.1–20.5 μmol/L)	25.2	8.7	6.5	15.5

ALP = alkaline phosphatase, ALT = alanine aminotransferase, AST = aspartate transaminase, GGT = gamma glutamyl transferase, TBIL = total bilirubin.

**Table 2:** Other laboratory tests.

Rule out diseases	Inspection	Inspection results
HBV	HBsAg	Negative
	HBeAg	Negative
HCV	Anti-HCV	Negative
CMV	CMV-IgM	Negative
	EBV-IgA	Negative
EBV	EBV-VCA-IgA	Negative
	EBV-IgG	Negative
	ANA	Negative
	ASMA	Negative
AIH	Anti-LKM antibody	Negative
	SLA	Negative
	IgG (7–16g/L)	11
	Anti-Smith antibody	Negative
	AMA-M2	Negative
AID	Anti-RNP antibody	Negative
	Anti-nucleosome	Negative
	AHA (Anti–histone protein)	Negative
PBC	Anti-sp antibody	Negative
	AFP (0–7ng/ml)	2.35
Hepatoma	CA 19–9 (0–30 U/ml)	16.01
	CEA (0–5 ng/ml)	2.15

AFP = alpha-fetoprotein, AID = autoimmune disease, AIH = autoimmune hepatitis, AMN-M2 = anti-mitochondrial antibody type 2, ANA = antinuclear antibody, ASMA = anti-smooth muscle antibody, CA19-9 = carbohydrate antigen 19-9, CEA = carcinoembryonic antigen, CMV = cytomegalovirus, EBV = Epstein-Barr virus, EBV-IgG = Epstein-Barr virus immunoglobulin G, EBV-VCA-IgA = Epstein-Barr Virus viral capsid antigen immunoglobulin A, HBsAg = hepatitis B surface antigen; hepatitis B virus e antigen, HBV = hepatitis B virus, HCV = hepatitis C virus, LKM = liver kidney microsomal, PBC = primary biliary cholangitis, RNP = ribonucleoprotein, SLA = soluble liver antigen.

**Table 3:** RUCAM score.

RUCAM score				
Hepatocellular injury	Score	Result		
		Huzhang	Muxiang	Huangqin, etc
1. Time to onset from the beginning of the drug/herb		+2	+2	+1
5–90 d (rechallenge: 1–15 d)	+2			
<5 or > 90 d (rechallenge: >15 d)	+1			
≤15 d (except for slowly metabolized chemicals: >15 d)	+1			
2. Course of ALT after cessation of the drug/herb		+2	+2	+0
Decrease ≥ 50% within 8 d	+3			
Decrease ≥ 50% within 30 d	+2			
No information or continued drug use	+0			
Decrease ≥ 50% after the 30th d	+0			
Decrease < 50% after the 30th d or recurrent increase	−2			
3. Risk factors		+1	+1	+1
History of alcohol consumption	+1			
No history of alcohol consumption	+0			
Age ≥ 55 yr	+1			
Age < 55 yr	+0			
4. Concomitant drug(s)/herb(s)		−2	−2	−2
None or no information	+0			
Concomitant drug/herb with an incompatible time to onset	+0			
Concomitant drug/herb with an compatible or suggestive time to onset	−1			
Concomitant drug/herb known as a hepatotoxin and with a compatible or suggestive time to onset	−2			
Concomitant drug/herb with evidence for its role in this case (positive rechallenge or validated test)	−3			
5. Search for alternative causes		+2	+2	+2
Group I (7 causes)				
HAV: anti-HAV-IgM				
HBV: HBsAg, anti-HBc-IgM, HBV-DNA				
HCV: anti-HCV, HCV-RNA				
HEV: anti-HEV-IgM, anti-HEV-IgG, HEV-RNA				
Hepatobiliary sonography/color Doppler sonography of liver vessels/endosonography/CT/MRC				
Alcoholism (AST/ALT ≥ 2)				
Acute recent hypotension history (particularly if underlying heart disease)				
Group II (5 causes)				
Complications of underlying disease(s) such as sepsis, metastatic malignancy, autoimmune hepatitis, chronic hepatitis B or C, primary biliary cholangitis or sclerosing cholangitis, genetic liver diseases				
Infection suggested by PCR and titer change for				
CMV (anti-CMV-IgM, anti-CMV-IgG)				
EBV (anti-EBV-IgM, anti-EBV-IgG)				
HSV (anti-HSV-IgM, anti-HSV-IgG)				
VZV (anti-VZV-IgM, anti-VZV-IgG)				
Evaluation of group I and II	+2			
All causes-groups I and II: reasonably ruled out	+1			
All causes of group I were ruled out	+0			
4 or 5 causes of group I ruled out	−2			
<4 causes of group I ruled out	−3			
An alternative cause is highly probable				
6. Previous hepatotoxicity of the drug/herb		+1	+0	+0
Reaction labeled in the product characteristics	+2			
Reaction published but unlabeled	+1			
Reaction unknown	+0			
7 Response to unintentional reexposure		+0	+0	+0
Doubling of ALP with the drug/herb alone, provided ALP below 2ULN before reexposure	+3			
Doubling of ALP with the drugs(s)/herbs(s) already given at the time of 1st reaction	+1			
Increase of ALP but less than ULN in the same conditions as for the 1st administration	−2			
Other situations	+0			
Total score for the case		6	5	2

ALP = alkaline phosphatase, ALT = alanine aminotransferase, AST = aspartate aminotransferase, CMV = cytomegalovirus, CT = computer tomography, EBV = Epstein-Barr virus, HAV = hepatitis A virus, HBc = hepatitis B core, HBsAg = hepatitis B antigen, HBV = hepatitis B virus, HCV = hepatitis C virus, HEV = hepatitis E virus, HSV = herpes simplex virus, MRC = magnetic resonance cholangiography, RUCAM = Roussel Uclaf Causality Assessment Method, ULN = upper limit of the normal range, VZV = varicella zoster virus.

**Table 4:** History of herbal medicine use by the patient.

Date of taking medicine	January 7, 2020; February 21, 2020; April 1, 2020; May 22, 2020; June 24, 2020	February 25, 2020	April 13, 2020	November 7, 2020	November 18, 2020	October 12, 2022
Chinese herbal medicine	Baihe	Baishao	Bichengqie	Biejia	Biejia	Banxia
	Baishao	Baizhi	haipiaoshao	Danshen	Chishao	Bohe
	Baizhu	Baizhu	Chuanlianzi	Honghua	Chuanlianzi	Chaihu
	Chuanxiong	Chaihu	Dahuang	Jinqiancao	Chuanxiong	Dangshen
	Danɡɡui	Chenpi	Foshou	Chuanqiong	Chuipencao	Duzhong
	Diyu	Chuipencao	Huanglian	Chuipencao	Danshen	Gancao
	Jiujiechangpu	Dihuang	walenɡzi	Fangfeng	Fanɡfenɡ	Ganjiang
	Puhuanɡ	Gancao	Wuzhuyu	Mabiancao	Honɡhua	Houpo
	Sanqi	Huangqi	Xiangfu	Shashen	Lianqiancao	Huangqin
	Shihu	Huangqin	Xiangyuan	Taoren	Mabiancao	Jiegeng
	Wuyao	Huzhang	Yanhusuo	Wuweizi	Shashen	Kuandonɡhua
	Xuanshen	Muxiang		Yujin	Shiwei	
	Yanhusuo	Sharen			Taoren	Kuxingren
	Zexie	Shengma			Xiakucao	Mahuan
		Yujin			Xianhecao	Qianhu
		Zhiqiao			Yujin	Shegan
						Tianma
						Zisugeng
						Ziwan

Treatment commenced on April 4 with intravenous infusion of 8 g N–acetylcysteine once daily (QD) and oral administration of 70 mg silybin capsules 3 times daily for detoxification and liver protection. Additionally, 456 mg polyenylphosphatidylcholine capsules (Essentiale® Capsules) were given orally 3 times daily to repair liver cell membranes as part of the hepatoprotective regimen.

**Figure 2.**
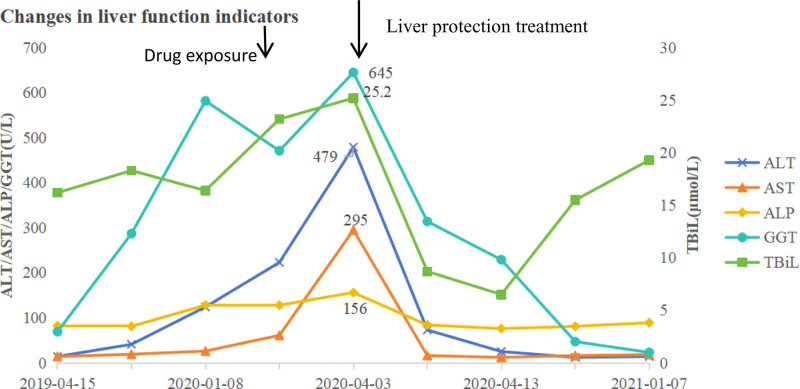
Changes in liver function indicators. ALP = alkaline phosphatase, ALT = alanine aminotransferase, AST = aspartate transaminase, GGT = gamma glutamyl transferase, TBIL = total bilirubin.

On April 8, follow-up liver function tests showed alanine aminotransferase 73 U/L, aspartate transaminase 16 U/L, alkaline phosphatase 84 U/L, and total bilirubin 8.7 umol/L, indicating that the patient’s liver function was improving and had returned to normal levels. The patient was discharged on April 14. During the 1-month follow-up on May 23, all liver function indicators were normal (Table 1, Fig. 2).

## 3. Discussion and conclusion

Traditional Chinese herbs have potential hepatotoxicity, and due to their widespread use, the incidence of liver injury ranks high in China. The updated RUCAM serves as a powerful diagnostic tool for DILI, providing a relatively objective assessment and finding widespread application in DILI cases (Table S1, Supplemental Digital Content https://links.lww.com/MD/R260).^[5]^ DILI is a diagnosis of exclusion. The patient was admitted to the hospital on April 3 after discovering elevated liver enzymes, and further tests were conducted. Upon admission, the patient was conscious and had no other underlying diseases except for a history of gastritis, thus ruling out shock and other serious diseases. Based on negative results for HBsAg and HCV-IgM, hepatitis B and C were ruled out. Negative results for CMV-IgM and EBV-IgA antibodies excluded cytomegalovirus and EBV infections. Negative results for ANA, anti-smooth muscle antibody, and anti-SLA antibodies ruled out autoimmune hepatitis and immune-related diseases. Additionally, abdominal CT showed normal liver and pancreas morphology, with no abnormal density in the liver parenchyma, ruling out organic lesions of the liver, gallbladder, and pancreas via CT. Combined with relevant tumor marker tests such as alpha-fetoprotein, liver tumors were also ruled out. Furthermore, the patient denied alcohol consumption, thus ruling out alcoholic liver disease. After ruling out various diseases, we considered drug-related or other factors causing liver injury. Therefore, we inquired about the patient’s medication history over the past 6 months and retrieved the prescription records. It was found that the patient had intermittently taken Chinese herbal medicine since January 2020 (Table 4). The formula, which included Baihe and Xuanshen, was continued until June. However, the patient’s liver function returned to normal after treatment on April 13, so this formula could be ruled out. Then, the patient took a new formula containing Huzhang and Muxiang for 14 days. Subsequently, the patient developed time-related liver function abnormalities. According to the updated RUCAM, the score for Huzhang was 6, indicating a highly possible causal relationship. The scores were 5 for Muxiang, and 2 for other herbs, including Huangqin (Table 3). This leads us to strongly suspect that Huzhang-induced liver injury.

Muxiang is the dried root of the Asteraceae plant Aucklandia lappa Decne. According to data from the https://www.bic.ac.cn/TCMSTD/ (TCM-STD) and Livertox,^[6]^ no hepatotoxicity associated with wood fragrant has been identified. The sesquiterpenoid compounds in Muxiang, including Aucklandin and Dehydroaucklandin, exhibit anti-inflammatory and antioxidant effects, demonstrating potential hepatoprotective properties.^[7]^ Additionally, Kun Song et al demonstrated that Muxiang influences the Nrf2/HO-1 pathway, activating the antioxidant stress response system to improve liver damage in rats.^[8]^ Lei Zhao et al studied the protective effects of Muxiang on lipopolysaccharide-induced liver damage in human normal Lo2 liver cells.^[9]^ In summary, for this case, we should exclude the influence of Muxiang.

Next, we move on to Huzhang (Polygoni Cuspidati Rhizoma et Radix), which refers to the dried rhizomes and roots of the plant Polygonum cuspidatum Sieb. et Zucc. According to the Traditional Chinese Medicine Systems Pharmacology Database and Analysis Platform (TCMSP, https://www.tcmsp-e.com/browse.php?qc=herbs) and the Swiss Target Prediction Database (http://www.swisstargetprediction.ch/index.php), and other databases, indicate that Huzhang contains anthraquinone compounds such as emodin. The Chinese Pharmacopoeia specifies that Huzhang contains at least 0.6% emodin, a component also present in Heshouwu (Polygonum multiflorum radix) and rhubarb (Rhei Radix et Rhizoma).^[10]^ The emodin content in raw rhubarb and processed rhubarb is 0.401% and 0.669%, respectively.^[11]^ The emodin content in rhubarb and Huzhang should be comparable. Free emodin is primarily present in the liver,^[12,13]^ and long-term high-dose emodin has hepatotoxicity and cytotoxicity.^[14]^ Current research on emodin primarily focuses on its mechanism of inducing mitochondrial damage in the liver, as well as inducing endoplasmic reticulum stress and disrupting bile acid metabolism in hepatocytes.^[15]^ A study utilizing L02 cells revealed through quantitative proteomics and Western blotting that emodin interferes with oxidative phosphorylation pathways by inhibiting the function of all mitochondrial respiratory chain complexes, thereby reducing ATP synthesis, and ultimately inducing hepatocyte apoptosis.^[16]^ Jingzhuo Tian et al demonstrated through animal studies that long-term intake of emodin affects the farnesoid X receptor - retinoid X receptor-cholesterol – cholesterol–7α–hydroxylase pathway, promoting hepatic secretion of primary bile acids. Biliary stasis subsequently induces liver damage.^[17]^ Emodin may also interfere with hepatic glutamine metabolism, activate stress responses, accelerate hepatic stellate cell senescence, and potentially increase the risk of liver fibrosis during long-term medication use.^[18]^ This patient developed liver injury after taking 15 g of Huzhang twice daily for 2 weeks. Although limited records of hepatotoxicity exist for Huzhang in databases, its emodin content is comparable to that of known hepatotoxic Chinese medicinal herbs. Consequently, we must not overlook its potential risks. This case concerns a 72-year-old female patient, an elderly individual with no underlying conditions other than chronic gastritis. She has a history of right shoulder surgery and cholecystectomy, factors that may collectively increase susceptibility to DILI. Decreased activity of hepatic metabolic enzymes and reduced hepatic and renal blood flow in the elderly may lead to delayed drug clearance and drug accumulation, thereby exacerbating hepatotoxic reactions. A Spanish study found that patients over 65 years of age accounted for 33% of DILI cases.^[19]^ Age may influence a patient’s sensitivity to medications, and this possibility may also be related to the long-term use of multiple medications in elderly patients.^[20]^ A study suggested that the risk of acute DILI increases 6-fold when 2 or more hepatotoxic medications are used.^[21]^ Aging is also associated with immune function, and impaired immunity can be considered 1 of the risk factors for DILI.^[22]^ Surgical trauma may induce a systemic inflammatory response or alterations in immune function, thereby affecting drug metabolism and hepatic repair capacity. A case-control study suggests that particular attention should be paid to patients with hyperlipidemia, cardiovascular disease, a history of liver disease, and a history of surgery, as these factors may increase the incidence of DILI.^[23]^ This suggests that we need to be cautious about the occurrence of liver injury in the future when dealing with patients with a history of surgery in terms of medication. Through this case analysis, the systematic tracing of medication history, the exclusion of other diseases, and the combination of the updated RUCAM demonstrated the diagnostic process of DILI. However, there are still some limitations in this case, such as the lack of detection of hepatitis E, in addition to the fact that the result of the updated RUCAM was “probable” rather than “certain”, indicating that more epidemiological and mechanistic studies are needed to confirm the causal relationship between the Huzhang and liver injury. In the future, clinical studies with larger samples should be carried out to clarify the true incidence of hepatotoxicity, the dose-effect relationship, and the individual susceptibility factors of Huzhang, so as to provide a more solid evidence base for the safe and rational use of Chinese herbal medicines in clinical practice.

This study reveals the clinical characteristics and mechanisms of the hepatotoxic herbs through a case of DILI associated with Huzhang. In the elderly and with a history of surgery, patients taking potentially hepatotoxic herbs such as Huzhang are assessed for individual risk. During its use, strict limits on dosage and duration of treatment should be enforced, and regular monitoring of liver function is essential. And the updated RUCAM could be a tool to help diagnose DILI in the clinic. It helps us to identify drugs that may cause liver injury and alert doctors to use them carefully in future medications. This aims to reduce the risk of liver injury in patients.

## Acknowledgments

The authors would like to thank the patient involved in this study. This study was funded by a grant from the Jiangsu Provincial Drug and Food Administration (2023084). And this study was approved by the Nanjing Hospital of Chinese Medicine ethics committee (Ethics Case Number: KY2025011). Written informed consent was obtained from the patient for publication of this case report and any accompanying images.

## Author contributions

**Writing – original draft:** Xinyan Liang.

**Data curation:** Xiu Yang.

**Writing – review & editing:** Xiu Yang.

**Formal analysis:** Tianjiong Luo.

**Supervision:** Tianjiong Luo, Wenliang Dun.

## Supplementary Material



## References

[1] Skat-Rørdam J Lykkesfeldt J Gluud LL Tveden-Nyborg P. Mechanisms of drug-induced liver injury. Cell Mol Life Sci. 2025;82:213. 40418327 10.1007/s00018-025-05744-3 PMC12106265

[2] Chinese Association of Pharmaceutical Biotechnology, Drug-Induced Liver Injury Prevention and Treatment Technical Committee; Chinese Medical Association, Hepatology Branch, Drug-Induced Liver Disease Working Group. Chinese guidelines for the diagnosis and treatment of drug-induced liver injury (2023 Edition). Chin J Hepatol. 2023;31:355–84.

[3] Shen T Liu Y Shang J. Incidence and etiology of drug-induced liver injury in Mainland China. Gastroenterology. 2019;156:2230–41.e11. 30742832 10.1053/j.gastro.2019.02.002

[4] Chinese Medical Association, Chinese Medical Association Journal, Chinese Medical Association Hepatology Branch Drug-Induced Liver Disease Working Group. Guidelines for primary care diagnosis, treatment, and management of drug-induced liver injury in China (2024). Chin J Gen Pract. 2024;23:813–30.

[5] Jing J Teschke R. Traditional Chinese medicine and herb-induced liver injury: comparison with drug-induced liver injury. J Clin Transl Hepatol. 2018;6:57–68. 29577033 10.14218/JCTH.2017.00033 PMC5863000

[6] LiverTox: Clinical and Research Information on Drug-Induced Liver Injury. National Institute of Diabetes and Digestive and Kidney Diseases; 2012–. Herbal and Dietary Supplements; 2025. 31643176

[7] Yang W Xiaoxiao F Jun Y. Research progress on terpenoid components and pharmacological effects of wood fragrance. Chin J Trad Chin Med. 2020;45:5917–28. 10.19540/j.cnki.cjcmm.20200903.601 33496131

[8] Shen S Donghui W Rongli Q. Study on the protective effect of wood fragrance ethanol extract on lipopolysaccharide-induced liver injury in rats. Chin J Trad Medi. 2023;38:6020–3.

[9] Lei Z Dang Y Zhaoyue D. Chemical constituents of Sichuan wood fragrant and its anti-lipopolysaccharide-induced liver injury effects. Chinese Herbal Medicines. 2025;56:4556–64.

[10] Committee National Pharmacopoeia. Pharmacopoeia of the People’s Republic of China [M] Part 1. China Medical Science Press; 2020:217–8.

[11] Jianhua L Sumei Z Xinhou Z. Determination of rhein content in different processed forms of rhubarb by HPLC. Chin J Vet Drugs. 2006;40:9–12.

[12] Yang J Wang Y Cai X. Comparative pharmacokinetics and tissue distribution of polydatin, resveratrol, and emodin after oral administration of Huzhang and Huzhang-Guizhi herb-pair extracts to rats. J Ethnopharmacol. 2024;318(Pt B):117010. 37557937 10.1016/j.jep.2023.117010

[13] Lin SP Chu PM Tsai SY Wu MH Hou YC. Pharmacokinetics and tissue distribution of resveratrol, emodin and their metabolites after intake of Polygonum cuspidatum in rats. J Ethnopharmacol. 2012;144:671–6. 23069945 10.1016/j.jep.2012.10.009

[14] Cui Y Chen LJ Huang T Ying JQ Li J. The pharmacology, toxicology and therapeutic potential of anthraquinone derivative emodin. Chin J Nat Med. 2020;18:425–35. 32503734 10.1016/S1875-5364(20)30050-9

[15] Wang Y Zhao M Li B Geng X. Advances in the mechanism of emodin-induced hepatotoxicity. Heliyon. 2024;10:e33631. 39027614 10.1016/j.heliyon.2024.e33631 PMC11255441

[16] Lin L Liu Y Fu S Qu C Li H Ni J. Inhibition of mitochondrial complex function-the hepatotoxicity mechanism of emodin based on quantitative proteomic analyses. Cells. 2019;8:263. 30897821 10.3390/cells8030263 PMC6468815

[17] Jingzhuo T Lianming W Yan Y. Mechanistic study on long-term rhein-induced liver injury and bile acid metabolism. Chin J Trad Chin Med. 2025;50:3079–87. 10.19540/j.cnki.cjcmm.20250103.704 40686176

[18] Chen L Liang B Xia S. Emodin promotes hepatic stellate cell senescence and alleviates liver fibrosis via a nuclear receptor (Nur77)-mediated epigenetic regulation of glutaminase 1. Br J Pharmacol. 2023;180:2577–98. 37263753 10.1111/bph.16156

[19] Lucena MI Andrade RJ Kaplowitz N; Spanish Group for the Study of Drug-Induced Liver Disease. Phenotypic characterization of idiosyncratic drug-induced liver injury: the influence of age and sex. Hepatology. 2009;49:2001–9. 19475693 10.1002/hep.22895

[20] Li X Tang J Mao Y. Incidence and risk factors of drug-induced liver injury. Liver Int. 2022;42:1999–2014. 35353431 10.1111/liv.15262

[21] de Abajo FJ Montero D Madurga M García Rodríguez LA. Acute and clinically relevant drug-induced liver injury: a population-based case-control study. Br J Clin Pharmacol. 2004;58:71–80. 15206996 10.1111/j.1365-2125.2004.02133.x PMC1884531

[22] Lucena MI Sanabria J García-Cortes M Stephens C Andrade RJ. Drug-induced liver injury in older people. Lancet Gastroenterol Hepatol. 2020;5:862–74. 32818465 10.1016/S2468-1253(20)30006-6

[23] Kong X Guo D Liu S Zhu Y Yu C. Incidence, characteristics and risk factors for drug-induced liver injury in hospitalized patients: a matched case-control study. Br J Clin Pharmacol. 2021;87:4304–12. 33948989 10.1111/bcp.14847

