# Supplementary material for: A case report and a literature review of drug-induced liver injury caused by Huzhang according to the updated RUCAM
Source: Medicine (Baltimore). 2026 Jan 30;105(5):e47385. doi: 10.1097/MD.0000000000047385 (PMC12863832; doi:10.1097/MD.0000000000047385)
Supplement: Supplementary file 1 [file medi-105-e47385-s001.doc]

Table S1 RUCAM causal assessment scale

| **medicinal ：** | **initial ALT>** | **initial ALP>** | **R value = (ALT/ULN)/| (ALP/ULN)|=** |  |
| --- | --- | --- | --- | --- |
| Type of liver injury: hepatocellular type (≥5.0), cholestatic type (≥2.0), mixed type (≥2.0 or <5.0) |  |  |  |  |
| Cholestatic or mixed type | | Cholestatic or mixed type | | appraise |
| 1. Time from medication to onset | First medication | Repeat medication | First medication | Repeat medication |
| ○ Open the bottle of the drug |  |  |  | grade |
| ● point out | 5~90 d | 1~15 d | 5~90 d | 1~90 d |
| ● suspicious | <5 or> 90 d | >15 d | <5 d or> 90 d | >90 d |
| ○ From the start of discontinuing the drug |  |  |  |  |
| ● suspicious | <15 d | <15 d | >30 d | <30 d |
| Note: If liver injury occurs at the beginning of medication, or if the drug is>35 days (liver cell type)>30 days (cholestatic type), then consider that the liver injury is unrelated to the drug and should not continue with RUCAM scoring | | | | |
| 2. Course of the disease | ALT varies between peak and ULN | | ALP (or Tbil) varies between peak and ULN | |
| ○ After discontinuation of the drug |  |  |  |  |
| ● High alert | 8d drop>50% | not applicable |  | +3 |
| ● suspicious | 30 days within a 50% drop | 180 d within decline>50% |  | +2 |
| ● Not suspicious | not applicable | 180 d within decline>50% |  | +1 |
| ● No conclusion | No data, or 30d later decline>50% | Not variable, increased or no data |  | 0 |
| ● Drug action related | A drop>50% or two increases after 30 days | This index is not applicable |  | -2 |
| ○ If the medication is continued |  |  |  |  |
| ● No conclusion | Any of the above occurs | Any of the above occurs |  | 0 |
| 3. Risk factors |  |  |  |  |
| ○ Alcohol consumption or pregnancy | drink | Alcohol or pregnancy (either one) |  |  |
|  | have | have |  | +1 |
|  |  |  |  | 0 |
| ○ age |  |  | >55 years old | +1 |
|  | <55 years old |  | <55 years old | 0 |
| 4. Comorbidities |  |  |  |  |
| ○ No concomitant medication, or no data, or concomitant medication does not match the onset time |  |  |  | 0 |
| ○ The time of onset coincides with the administration of the drug |  |  |  | -1 |
| ○ There is hepatic toxicity associated with the drug, and the onset time suggests or corresponds to it |  |  |  | -2 |
| ○ Evidence of hepatic injury with the drug (empty positive re-stimulus response, or clear association with hepatic injury and typical warning signs) |  |  |  | -3 |
| 5. Etiology of laboratory findings |  |  |  |  |
| Group I (type of etiology) |  |  |  |  |
| ○ Acute hepatitis type | Anti-HAV (-) IgM (+) or HBV triple positive | HbsAg and/or HBeAg (-) IgM (+) or HCV infection | Anti-HCV (+) and/or HCV RNA (+), accompanied by corresponding clinical manifestations* |  |  |  |  |
| ○ Biliary obstruction | confirmed by imaging |  |  |  |  |
| ○ Alcoholism| History of vinegar drinking and AST/ALT>2 |  |  |  |  |
| ○ Recent history of schistosomiasis, shock or liver ischemia | Within 2 weeks of onset |  |  |  |  |
| Group II (type of etiology) |  |  |  |  |
| ○ Hepatitis B, hepatitis C, tuberculous peritonitis, bacterial, chronic bronchitis or cholangitis | primary biliary cirrhosis (PBC) or primary sclerosing cholangitis (PSC) and other underlying diseases |  |  |  |  |
| ○ Clinical symptoms or laboratory tests suggest acute CMV, EBV or HSV infection |  |  |  |  |
| All causes were excluded from group I |  |  |  | +2 |
| All etiologies in group I were excluded |  |  |  | +1 |
| Four of the five causes in group I were excluded |  |  |  | 0 |
| There were fewer than 4 causes in group I of exclusion |  |  |  | -2 |
| ● High probability of non-pharmacological liver injury |  |  |  | -3 |
| 6. Diagnostic basis of drug-induced liver injury |  |  |  |  |
| ○ There is a history of drug use and it is indicated in the instructions |  |  |  | +2 |
| ○ Liver injury reactions are not specified in the instructions, but have been reported |  |  |  | +1 |
| ○ The hepatotoxicity response is unknown |  |  |  | 0 |
| 7. Reactions to re-administration |  |  |  |  |
| ○ positive | ALT increased by two times after repeated use of the drug | After using the drug again alone, ALP (or Tbil) increased by two times |  | +3 |
| ○ suspicious | Alleviation of ALP (or Tbil) by a factor 2 increase with concomitant use of another drug during recurrent and initial liver injury |  |  | +1 |
| ● negative | ALT was elevated again after a single use of the drug, but below ULN | After repeated use of the drug alone, ALP (or Tbil) increased, but below ULN |  | -2 |
| ○ No further medication or no determination |  |  |  | 0 |
| Overall assessment: >8 points very likely; 6-8 points probable; 3-5 points possible; 1-2 points unlikely; ≤0 points excluded | | | | |
| Note: ALT (Alkaline Lysyl Tryptase); ALP (Alkaline Phosphatase); ULN (Upper Limit of Normal); Tbil (Total Bilirubin); HBV (Hepatitis B Virus); IgM (Immunoglobulin M); HCV (Hepatitis C Virus); RNA (Ribosomal Acid); AST (Aspartate Aminotransferase); CMV (Cytomegalovirus); EBV (Epstein-Barr Virus); HSV (Herpes Simplex Virus); | | | | |
